# Smart Building: Decision Making Architecture for Thermal Energy Management

**DOI:** 10.3390/s151127543

**Published:** 2015-10-30

**Authors:** Oscar Hernández Uribe, Juan Pablo San Martin, María C. Garcia-Alegre, Matilde Santos, Domingo Guinea

**Affiliations:** 1 Advanced Technology Centre (CIATEQ, A.C.), Av. del Retablo 150 Col. Constituyentes Fovissste Querétaro, Qro. 76150, Mexico; E-Mail: oscar.hernandez@ciateq.mx; 2 Centre for Automation and Robotics (UPM-CSIC), Arganda del Rey, Madrid 28500, Spain; E-Mails: jp.sanmartin@csic.es (J.P.S.M.); domingo.guinea@csic.es (D.G.); 3 Department of Computer Architecture and Automatic Control, Faculty of Informatics, Complutense University, Madrid 28040, Spain; E-Mail: msantos@ucm.es

**Keywords:** decision-making architecture, distributed sensor network, dynamic thermal barrier, thermal energy, smart building, nZEB

## Abstract

Smart applications of the Internet of Things are improving the performance of buildings, reducing energy demand. Local and smart networks, soft computing methodologies, machine intelligence algorithms and pervasive sensors are some of the basics of energy optimization strategies developed for the benefit of environmental sustainability and user comfort. This work presents a distributed sensor-processor-communication decision-making architecture to improve the acquisition, storage and transfer of thermal energy in buildings. The developed system is implemented in a near Zero-Energy Building (nZEB) prototype equipped with a built-in thermal solar collector, where optical properties are analysed; a low enthalpy geothermal accumulation system, segmented in different temperature zones; and an envelope that includes a dynamic thermal barrier. An intelligent control of this dynamic thermal barrier is applied to reduce the thermal energy demand (heating and cooling) caused by daily and seasonal weather variations. Simulations and experimental results are presented to highlight the nZEB thermal energy reduction.

## 1. Introduction

Buildings produce high CO_2_ emissions as they consume almost 40% of worldwide energy, and a remarkable percentage of this energy is used for achieving thermal comfort conditions, both in heating and cooling. Thermal comfort is one of the first priorities, as it represents around 65% of building energy consumption. This point is highlighted by government policies and tax incentives aimed at efficient energy management to reduce CO_2_ emissions [1,2]. The European Parliament has adopted guidelines that require public buildings after 2019 and new buildings after 2021 to comply with the regulations to be certified as nearly zero energy buildings (nZEB). In these buildings the annual net balance between energy production and consumption should be close to zero [3].

Solar energy is the most widely renewable energy all over the World [4]. Solar power is sufficient to cover the thermal comfort demands in medium and low latitude regions. However, the variations in solar radiation, including day-night and seasonal cycles, require thermal energy storage systems (TES) to adjust the thermal demand to the energy production.

Another way of storing energy in a building is by means of a wall envelope. Wherever there is a difference in temperature the heat flows naturally from a warmer to a cooler space. During the summer, heat moves from the outside into the building, and the opposite happens in winter. The excess of heat in summer and the heat losses in winter need to be managed to maintain thermal comfort at low cost. Even more, the heating and air conditioning loads increase by daily and seasonal outdoor temperature variations. In buildings, the wall envelope has been proved to be a key factor to reduce heating/cooling loads [5]. To make better use of the thermal energy, some scientific publications have studied the thermal demand under different weather conditions, applying new approaches such as the inclusion in the walls of encapsulated phase change materials (PCMs), cellulose, expanded polystyrene or vegetation as roof top layers [6,7,8]. Other works report the inclusion of fluid-based heat exchange circuits embedded in the structure by using TES [9,10,11,12]. In [13], a microencapsulated PCM is added to concrete walls to substantially reduce and delay the thermal load on the building, trying to minimize the energy flow and, at the same time, reach the desired indoor temperature. Following the approach proposed in [14], closely related works are presented in [15,16]. In these cases, a full 3D finite element model focused on the thermal barrier (TB) is presented, where a novel control system is used to simulate real-working conditions. A semi-dynamic model of an active pipe-embedded building envelope is presented in [17], using three different composite walls.

These works prove that a better design of the external walls with a thermal barrier, with a multi-layer structure composed of at least three layers (insulation-concrete-insulation), provide a significant reduction in the energy consumption and a much better use of the energy to achieve thermal comfort in buildings.

Therefore, one of the main contributions of this paper is to propose the use if a dynamic thermal barrier. This element, integrated with a solar collector and a low enthalpy geothermal system, has been designed, simulated and implemented in an nZEB prototype building, located in Madrid (Spain). The novelty of current approach is the way it manages the fluid flow between the different thermal systems of the building, including the heating transfer to the thermal barrier. The control of the energy flow between these three systems allows a better management of the thermal energy in the building to meet users comfort demand.

On the other hand, currently energy systems are composed of numerous small-scale distributed power generation sources and storage systems, interconnected in micro-grids, with conventional centralized energy generation modules [18]. Therefore, electric companies are interested in controlling and monitoring all these types of heterogeneous devices connected to the electrical and energy infrastructures [19]. In this context, the Internet of Things (IoT) has emerged as a way to improve the conventional strategies for heating/cooling devices management [20], where pervasive sensors are used for the automatic control of the energy components, based on user behaviours, profiles, and preferences [21]. This new paradigm improves the efficiency of the thermal energy demand by the experience gathered from the customers. Machine learning algorithms or soft computing techniques are integrated to manage the interconnections among different sources including data provided by user context [22,23,24]. These energy monitoring and control schemes are related to the sensing, processing, networking devices and advanced techniques [25,26].

Following this approach, in this work a decision-making architecture to manage the capture, storage and use of solar thermal energy is presented. It includes the monitoring and control of the roof solar system, the ground heat exchanger, and the dynamic envelope (thermal barrier) using virtual sensors. These systems are integrated in the nZEB prototype that takes into account the occupant behaviours by means of home and user context virtual sensors. Intelligent control (fuzzy logic) and context information are used to represent and integrate knowledge for controlling the dynamic envelope to reduce the thermal energy use for heating and cooling.

The main contributions of current work is summarized as follows: the analysis of the solar collector simulation and experimental results for different parameters; the flow of the thermal energy when a dynamic thermal barrier is integrated in a building; the whole thermal energy transfer model of the experimental building; the developed of an architecture to control, using virtual sensors, context information, and computational intelligence techniques (decision trees and fuzzy logic), the thermal flow between the energy subsystems of the nZEB building; experimental validation in the building prototype by temperature and presence sensor measurements and wall thermal images.

The paper is organized as follows: in the next section the modelling and simulation of the three proposed thermal subsystems are presented. In Section 3, the sensor network and the layers of the decision making architecture proposed to control the thermal flow in the nZEB building are described. Section 4 shows the results of the tests carried out in the prototype of a nearly Zero Energy Building. Conclusions end the paper.

## 2. Thermal Energy Acquisition, Storage and Transfer Systems

In this section, the three thermal subsystems that have been designed and integrated to better use the thermal energy in a building are described in detail. These are:

- The solar collector (located in the roof)
- The low enthalpy geothermal heat store (ground heat exchanger)
- The dynamic thermal barrier (walls)

**Figure 1:**
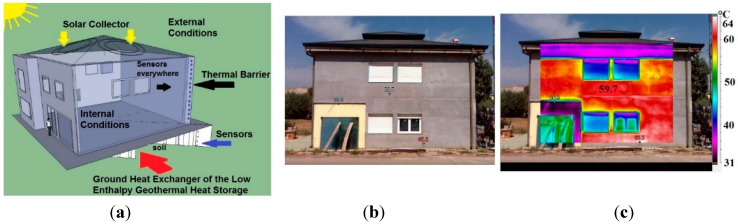
(**a**) Scheme of the nZEB building with the solar collector, the low enthalpy geothermal subsystem and the dynamic thermal barrier; (**b**) Building prototype; (**c**) Thermal image of the nZEB South façade.

They are interconnected by the hydraulic circuit (Section 2.4) that controls the fluid flow. This proposal has been implemented and tested in a real prototype building. The experimental nZEB building is located at the Centre for Automation and Robotics (CAR_CSIC_UPM), in Madrid (Spain). The building and the thermal subsystems are presented in Figure 1.

### 2.1. Thermal Solar Energy Collector

**Figure 2:**
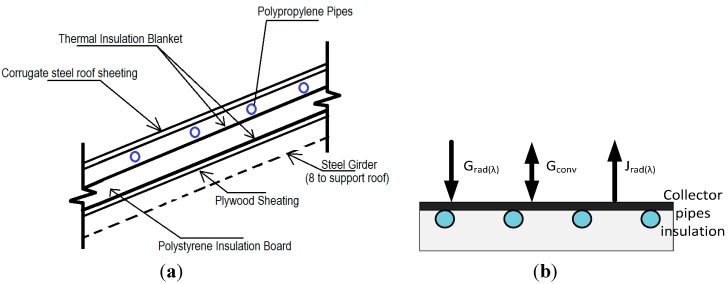
(**a**) Thermal solar collector scheme; (**b**) Energy balance in the collector surface.

A thermal solar energy collector has been integrated in the metal roof of the building. The roof is composed of an external layer of corrugated steel, shaped as a square hip roof. Two polypropylene (PP) tubes circuits are fixed to the corrugated steel’s inner face. The thermal energy captured by the metal layer is transferred to a water-glycol fluid flowing in the pipes, supported by a thermal multilayer insulation blanket and a polystyrene insulation board (Figure 2a).

The collector is responsible for the capture of the solar energy and plays a fundamental role in the energy balance of the building. A model based on the finite element simulation software Comsol Multiphysics [27] has been developed to characterize the collector behaviour. It is divided in three zones that correspond to the different parts of the roof: the collector surface, where heat transfer processes between environment and collector occur; the pipes, where fluid flows, and the bottom insulation layer.

The thermal energy captured by the collector surface is transferred to the fluid, to be transported where it is demanded. Therefore, the energy balance in the collector layer determines the amount of solar energy that can be absorbed by the fluid, *i.e.*, the solar energy available for use.

The thermal balance in the collector is shown in Figure 2b and is given by Equation (1):

(1) Q=Grad(λ)+Gconv−Jrad(λ) [Wm2]

where G_rad(λ)_ and J_rad(λ)_ represent the energy gains and losses due to the radiation processes, respectively, depending both on the external and the collector temperatures, and G_conv_ is the thermal flow generated by convection.

Radiation gain is defined by the total incident radiation, short and long-wave. The short-wave radiation (SR) corresponds to the direct solar radiation, meanwhile long-wave radiation (LR) includes those produced by all bodies surrounding collector that emit any radiation.

According to Stefan-Boltzmann’s equation, the radiation gain is formulated as follows:

(2) $$G_{rad(\lambda )}=\sigma \cdot \alpha_{\lambda l}\cdot f\left(\text{Ω}\right)\cdot T_{amb}^{4}+\alpha_{\lambda s}\cdot SR\approx \alpha_{\lambda s}\cdot SR$$

where *T_amb_* refers to the external temperature, and *α_λl_* and *α_λs_* (alpha values), represent the average absorptivity for the long and shortwave spectrums of the total incident solar radiation, including both beam and diffuse radiation; *σ* is the Stefan-Boltzmann constant, and *f(Ω)* is the view factor between long-wave emitter and the surface. For a quasi-horizontal inclination of the solar collector, *f(Ω)* reaches a value close to zero. Thus, long-wave radiation has been considered negligible compared to the short wave one.

Radiation losses are given by Equation (3):

(3) $$J_{rad(\lambda )}=\sigma \varepsilon_{\lambda l}T_{col}^{4}$$

where $\varepsilon_{\lambda l}$ is the emissivity of the solar collector and *T_col_* refers to its temperature.

Convective processes are described by Newton’s cooling law, Equation (4):

(4) $$G_{conv}=h\left(T_{amb}-T_{col}\right)$$

where h represents the convection coefficient, which is dependent on the type of media, gas or liquid, the flow properties such as velocity, viscosity and other flow and temperature dependent properties.

According to the energy balance, the total energy captured by the surface is presented in Equation (5):

(5) $$Q=\alpha_{\lambda s}\cdot SR+h\left(T_{amb}-T_{col}\right)-\sigma \varepsilon_{\lambda l}T_{col}^{4}$$

This energy balance is partially controlled by means of the optical properties of the surface, as the energy captured can be optimized using a high shortwave absorptivity and low long-wave emissivity material. This behaviour is simplified applying the Kirchhoff’s law (emissivity equals to absorptivity), and considering the surface as a grey body (optical properties independent on the wavelength). Finally:

(6) $$Q=\alpha (SR-\sigma T_{col}^{4})+h\left(T_{amb}-T_{col}\right)$$

The boundary conditions of this external capture surface are obtained from Equation (6). A high value of the absorptivity optimizes the capture of energy when the solar radiation is higher than the radiation losses. That is, for certain given external conditions, the optical characterization of the collector is a relevant factor for its thermal performance.

**Figure 3:**
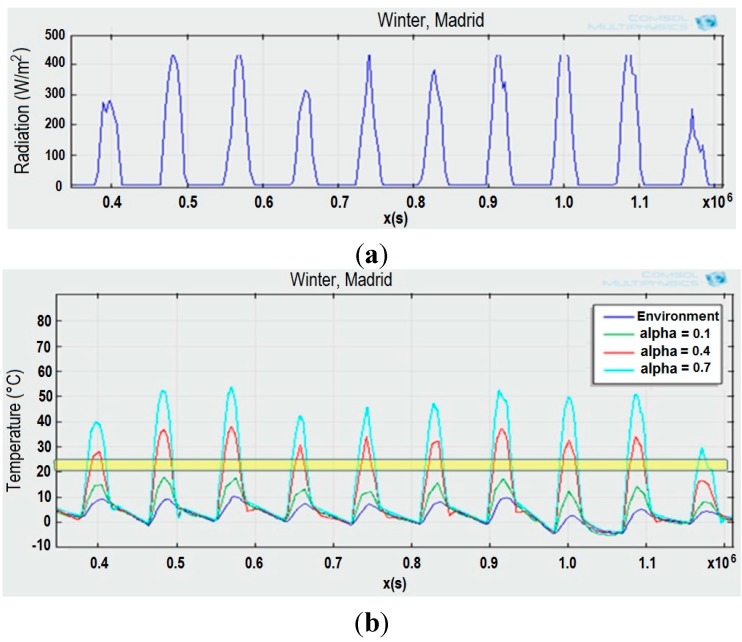
(**a**) Solar radiation in Madrid (Spain) in winter (10 days); (**b**) Temperature for different roof solar collector alpha values.

The behaviour of the solar collector has been analysed carrying out a sensitivity analysis, comparing the average collector surface temperature for different values of absorptivity, 0.1, 0.4 and 0.7 (Figure 3). The weather conditions are obtained with the TRaNsient SYstem Simulation (TRNSYS) software [28] and correspond to a standard meteorological year in Madrid (Spain). The solar radiation in the collector is displayed in Figure 3a. The simulation is carried out for a period of 10 winter days with a sampling of one hour. The results of the simulation are represented in Figure 3b. The temperature within the yellow band corresponds to the comfort band, 20 °C to 25 °C. Only when the collector temperature is higher than 25 °C, the energy captured covers the heating demand.

These results show the great influence of the absorptivity on the solar collector performance. Despite being in winter in Madrid (low temperatures), the average roof temperature reaches values above the comfort band when the wall absorptivity is greater than 0.4. The higher the absorptivity, the higher the available useful energy. Therefore, it is possible to use the thermal energy captured by the roof for heating purposes if the external surface is provided with optimal optical properties.

The simulation also shows how the available energy for cooling, with temperatures lower than 20 °C, is independent of the surface absorptivity. Therefore, the selection of a dark colour for the roof improves the thermal energy capture for heating purposes, without having a great impact on the thermal energy for cooling. The thermal circuit of the building transfers this solar energy captured in the roof either to the ground heat exchanger (Section 2.2), or to the dynamic envelope (Section 2.3).

### 2.2. Ground Heat Exchanger: Low Enthalpy Horizontal Ground Thermal Energy Source

**Figure 4:**
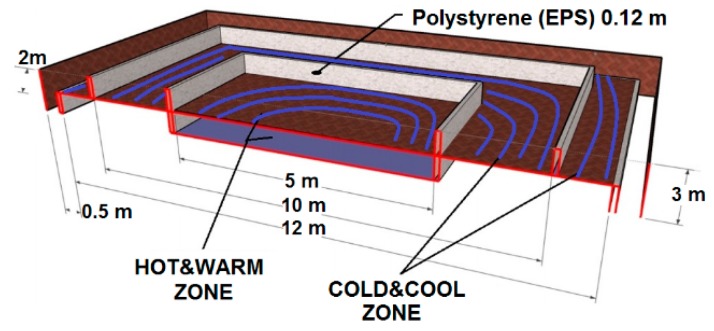
Cross section of the ground heat exchanger.

The ground storage system, made by concentric rings of polypropylene (PP) tubes, is located horizontally below the construction area (Figure 4). The soil is divided in four zones, namely: HOT, WARM, COOL and COLD, thermally independent of each other. The building perimeter is limited by a 10 m square, where hot and warm zones are located in concentric squares within the inner 5 m, and the rest is used as a cool zone. The cold zone is outside the building perimeter. These divisions were built by using expanded polystyrene (EPS) sheets of 2.4 m × 1.2 m × 0.12 m. These walls were placed vertically, as shown in the Figure 4, creating isolated areas to reduce the influence of weather conditions, such as the solar radiation, on the inner building [29].

The temperature ranges for the different thermal zones are shown in Table 1. The linguistic terms associated to these thermal zones of the ground heat exchanger will be used to control the thermal flow of the building.

**Table 1:** Linguistic terms associated to temperature ranges for the different zones, and pipes characteristics.

Temperature	Range	Characteristics
HOT	19–21 °C	2 PP pipes, ∅ 0.22 m, L 200 m, located 3 m underground, buried below the building
WARM	17–19 °C	2 PP pipes, ∅ 0.22 m, L 200 m, located 2 m underground, buried below the building
COOL	15–17 °C	3 PP pipes, ∅ 0.22 m, L 200 m, located 2 m underground, buried below the building
COLD	12–15 °C	2 PP pipes, ∅ 0.22 m, L 200 m, located 2m underground, buried outside the building

To summarize, the ground, characterized by a high thermal inertia, is used as a heat storage tank that accumulates the thermal energy (heating/cooling) according to the fluid temperature [30]. Absorbing/rejecting heat from/to the earth changes the soil temperature over years. This effect is shown in the results obtained in the experiments in the nZEB prototype. Nevertheless, the soil is used as a storage tank and do not consider this factor for multi-year operations. Thermal energy stored is used for heating/cooling the building envelope during cold/hot periods, on a diurnal or seasonal basis, and to keep the building in the comfort band along the year.

### 2.3. Dynamic Envelope: Thermal Barrier

The building envelope acts as a passive thermal barrier. This can be a key element in order to maintain a desired temperature in a building, particularly in cold climates, where the isolation layers of the walls can reduce the heating requirements. Besides, the right selection of the building envelope composition not only produces a better insulation but can also smooth the temperature peaks during the day-night cycle. In this work a dynamic thermal barrier with high thermal inertia is proposed. By using a controlled fluid flow in the inner zone of the exterior walls, the influence of the daily and seasonal outdoor temperature variations on the inner temperature of the building is reduced.

The wall is composed of three layers: two polystyrene insulation layers and, in the middle, a concrete layer. The parameters of the walls of the real nZEB building are listed in Table 2.

**Table 2:** Wall layers parameters.

Lightweight Concrete	Value	Units
Thickness	0.15	m
Density	1200	kg/m^3^
Specific Heat	800	J/kg·K
Thermal Conductivity	0.57	W/m·K
**POLYSTYRENE**		
Thickness	0.05	m
Density	35	kg/m^3^
Specific Heat	1450	J/kg·K
Thermal Conductivity	0.036	W/m·K
**nZEB**		
House Height	5	m
House Width	10	m
House Length	10	m
Windows	15	
Window Height	1	m
Window Width	1	M
Window Density	2500	kg/m^3^
Window Specific Heat	840	J/kg·K
Window Thermal Cond.	0.78	W/m·K
Initial Temperature	7.5	°C

The wall presents a notorious thickness, low thermal conductivity, and high density and specific heat. The combination of these properties provides an extremely high thermal inertia to the walls.

**Figure 5:**
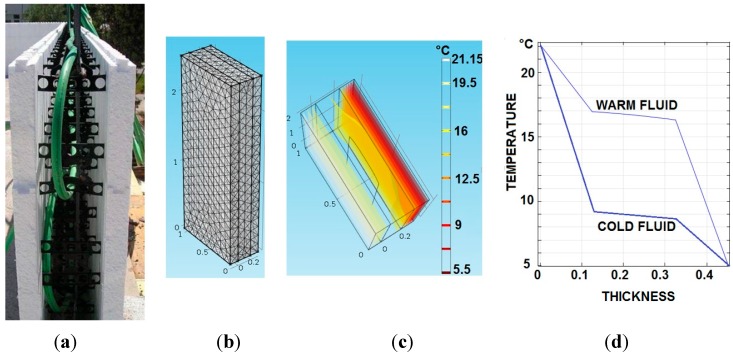
(**a**) PP pipes between two insulation layers of the wall; (**b**) Simulation of the finite elements wall model; (**c**) Wall isothermal distribution; (**d**) Temperature *vs* thickness.

PP tubes are embedded in the concrete layer, as shown in Figure 5a. Thus, a fluid can flow through them to increase the thermal capacity of the concrete layer, and therefore the insulation, by controlling its temperature.

The thermal performance of the dynamic thermal barrier has been modelled and simulated with COMSOL Multiphysics, Figure 5b, taking into account the energy transfer by conduction through the envelope. The initial values are: T_indoor = 22 °C and T_outdoor = 5 °C. Figure 5c shows the temperature distribution (isothermal contours), when a warm fluid at 17 °C is injected in the PP tubes of the thermal barrier. The indoor-outdoor temperature gradient with two different fluid temperatures (warm = 17 °C and cold = 9 °C) is shown in Figure 5d.

In the absence of PP tubes in the concrete layer, the temperature drop is due to the polystyrene layer because its thermal conductivity is less than 1/20 (0.028 W/m·K) compared to the thermal conductivity of the concrete wall. That is why the concrete wall is virtually isothermal at the indoor and outdoor average temperature. The pipes act as a temperature shield between the two insulation layers.

The temperature plateau of the dynamic thermal barrier for both warm and cold thermal energy flows represents the boundary condition. However, the temperature variation along the dynamic thermal barrier is low compared with the variation experienced along the two external layers. Therefore the thermal barrier reduces the thermal gradient and smooths the temperature variations. Thus, less thermal energy is required to reach an indoor comfort temperature.

**Figure 6:**
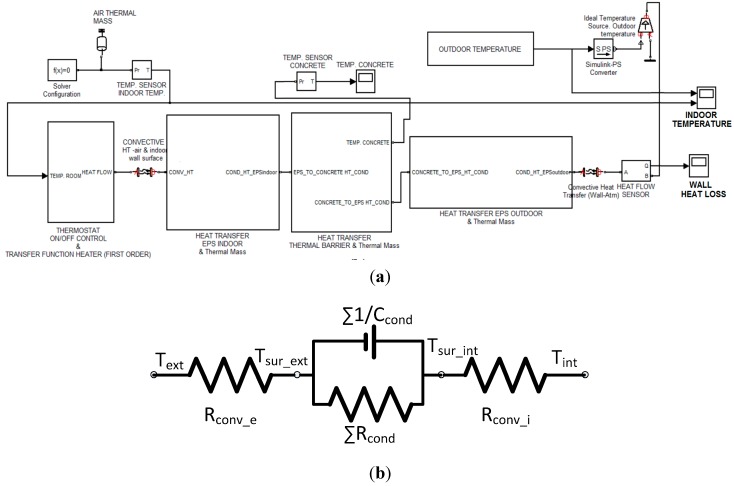
(**a**) Thermal energy transfer model of the experimental building; (**b**) Thermal equivalent circuit.

A thermal energy model of the building, that includes the dynamic envelope, has been developed using the computational software Matlab/Simulink (Figure 6a). The model represents the heat balance between building and environment through the envelope, including heat convection in both the inner and outer surfaces, and heat conduction through the envelope layers. These processes can be also represented by an equivalent circuit, Figure 6b.

Newton’s law of cooling has been used (Equation (7)), to represent the energy transfer in a surface in contact with a fluid by the convection process. In current case the fluids are the inner and external air:

(7) $$\frac{dQ}{dt}=Ah\Delta T$$

where h is the convection coefficient, which depends on external conditions such as wind velocity, humidity or air density, and surface characteristics like geometry or roughness. ΔT is the difference of temperature between the surface and the environment, and A the surface area.

The thermal resistance due to convection is defined in Equation (8):

(8) $$R_{conv}=\frac{1}{hA}$$

The energy transfer through the envelope is calculated with the heat transfer law (Equation (9)), and represents the energy variation through the thickness of the wall combining thermal resistance and thermal capacity:

(9) $$\frac{dQ}{dt}=k\frac{\partial^{2}T}{\partial x^{2}}-\rho C_{p}\frac{\partial T}{\partial t}$$

where k is the heat conductivity, C_p_ the heat capacity, and ρ the density of the material.

The thermal resistance due to conduction is formulated by Equation (10):

(10) $$R_{cond\_i}=\frac{L_{i}}{k_{i}A}$$

And the thermal capacity of each layer, Equation (11), is:

(11) $$C_{cond\_i}=\rho_{i}C_{pi}$$

As the envelope layers are connected in series, the equivalent resistance is obtained by adding up the individual resistance of each layer, and the opposite with the inverse equivalent capacity.

**Figure 7:**
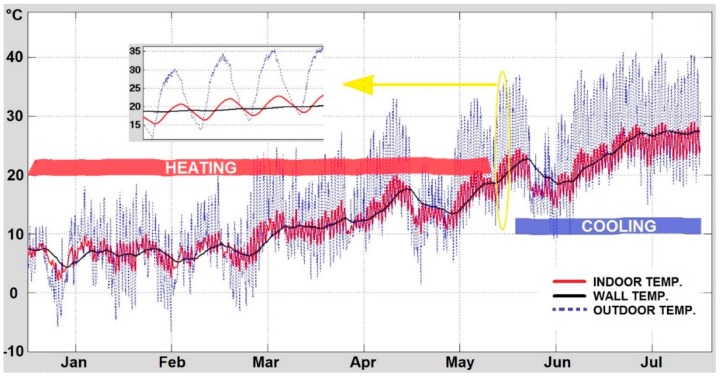
Thermal inertia, seasonal and daily temperatures. Outdoor measured temperature, and both indoor and wall simulated temperatures.

The simulation results show the influence of the external conditions on the indoor temperature of the building. In Figure 7, the indoor and wall temperatures obtained by the model are represented and compared with the measured external temperature, showing the influence of seasonal and daily weather variations, using the values displayed in Table 2. The indoor temperature is clearly affected by external weather conditions, presenting the same behaviour than the outdoor temperature, except for the delay and cushioning effect produced by the thermal inertia of the envelope (heat capacity). The temperature measured by the sensor located within the wall follows the same pattern but, in this case, it seems to be more independent of the day-night weather variations. It is clearly shown that in the first five months of the year an extra thermal energy (heating) is needed to reach the comfort temperature (red band). On the contrary, in the summer an extra thermal energy (cooling) is required for the building to keep the desired temperature (blue band).

**Figure 8:**
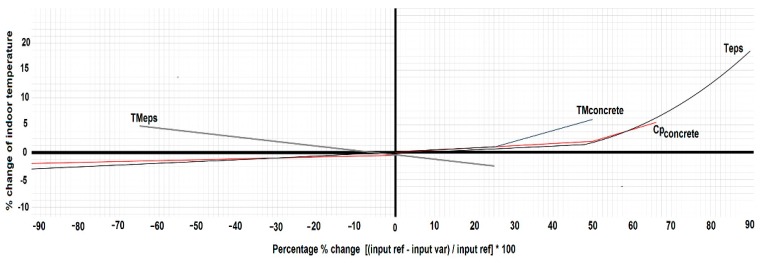
Effect of the building envelope parameters in the indoor temperature.

Besides, a sensitivity analysis was carried out, varying some of the wall parameters (Figure 8). The thermal mass is defined by three characteristics: specific heat, density, and thermal conductivity. For instance, a thermal mass change of 40% represents a similar variation of each property. For this study, the parameters analysed are: the thermal mass (TM_concrete and TM_eps), the concrete thermal capacity (Cp_concrete), and the polystyrene thickness layers (T_eps). The values of the indoor temperature while changing these parameters are shown in Figure 7. Remark that the thickness of the polystyrene layers (T_eps) is the most relevant parameter, when the wall is 90% thinner the indoor temperature increases around 20%. Similar behaviour is caused by changing the thermal mass of the concrete layer (TM_concrete), being the indoor temperature more affected by lower values. The variation of the temperature with polystyrene thermal mass (T_eps) confirms that the selected polystyrene parameters (Table 2) were adequate, when this parameter increases the temperature goes up but slowly. Finally, lower concrete thermal capacity (Cp_concrete) increases the indoor temperature. In all these cases the model response is as expected. The thickness of the polystyrene layer does not strongly influence the indoor temperature, opposite to the polystyrene thermal mass behaviour. In addition, it is easy to infer that if the thickness of both the concrete wall and the polystyrene increase, the temperature variations will be reduced, although a much better insulated building also increases the risk of overheating [31]. It is worth noting that Madrid is a city with a continental-Mediterranean climate, with average monthly temperatures of 5 °C in winter and 25 °C in summer.

To summarize, once the hydraulic circuit (Section 2.4) is connected, the fluid flows through the PP tubes embedded into the exterior wall (dynamic thermal barrier) and different temperature ranges are selected from the ground heat exchanger and solar collector, according to user profile, time and season.

### 2.4. Hydraulic Circuit

**Figure 9:**
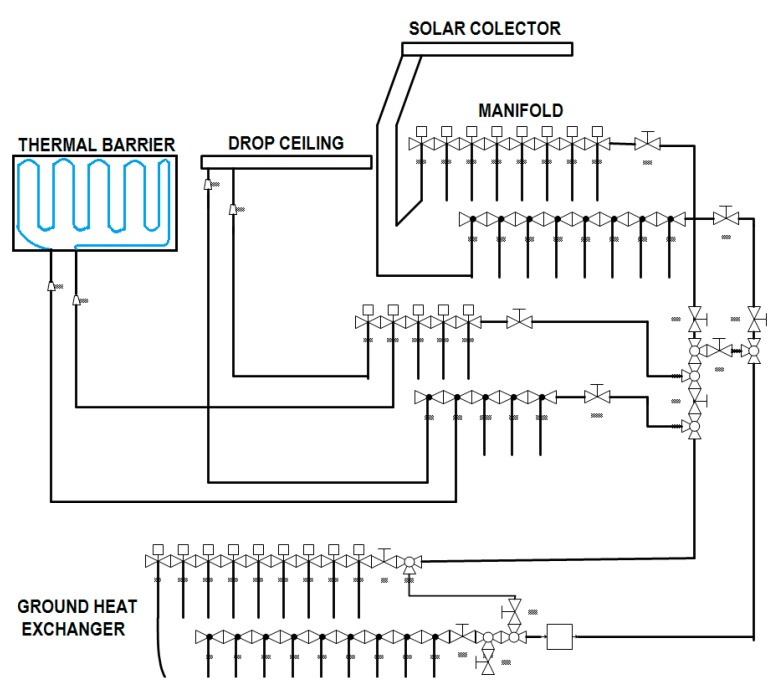
Hydraulic circuit and thermal subsystems.

Finally, a hydraulic circuit is implemented in the nZEB building to connect all the thermal subsystems previously described. The water-glycol fluid transport circuit is formed by the PP pipes connected to the roof solar collector, to the underground heat exchanger, and the ones of the dynamic thermal walls. Hot, warm, cool, or cold fluids flow through the PP tubes connecting the three different subsystems that act as sinks or sources of thermal energy, according to the demand and the availability. The scheme of the whole circuit is displayed in Figure 9.

The fluid recirculation is controlled by an input-output electro-valve, according to the fluid temperature and the thermal energy demand. Depending on the user requirements the system can either transfer the thermal energy (heating/cooling) from the roof solar collector to the façade or to the ground heat exchanger zones or, on the contrary, from the geothermal system to the façade. When the thermal energy captured is demanded by the user, it is directly sent to the dynamic thermal barrier to change the indoor temperature. Otherwise, the fluid is transferred to the ground heat exchanger for storage and later use.

## 3. Smart Building: A Perceptual and Decision-Making Architecture for Thermal Energy Management

**Figure 10:**
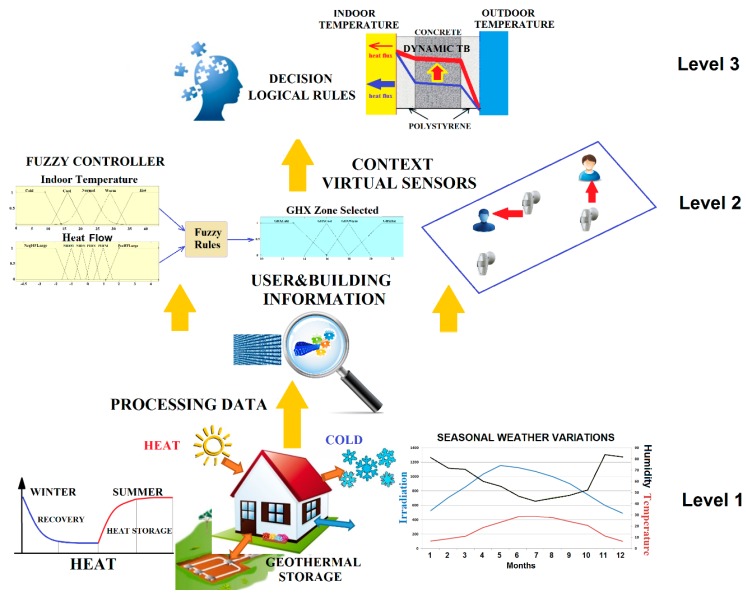
Three level perception and decision making architecture for thermal energy flow management.

A three level perception and decision-making architecture is designed for monitoring and controlling the thermal energy flow between the three thermal subsystems described in the previous section, according to the user comfort demand. The perception-control loops rely on the data acquired by the Internet of Things (IoT) nodes. In the nZEB prototype, the IoT perceptual nodes are presence, temperature, and flow sensors, and the IoT action nodes are linked to electro valves and pumps. Different interfaces are used for the visualization, information transfer to data bases, and updating of the shared memory. The three level perception and decision-making architecture is depicted in Figure 10.

The bottom level, level 1, corresponds to the physical sensor network that offers different communication channels, and is in charge of the raw data acquisition. In the level 2, a fuzzy rule based controller has been designed to manage the ground heat exchanger. Context information and virtual sensors are also integrated at this level. At the top level, level 3, the decision making system based on perceptions manages the thermal energy flows between the three subsystems, especially focused on the thermal barrier. The description of each of the three levels is presented in the following subsections.

### 3.1. Level 1: Sensor and Communication Layer

At this level, a network of physical sensors has been developed and implemented for signal acquisition, to monitor the thermal energy flow in the near Zero Energy Building. Three different IoT nodes are used, all of them with CAN-bus networking protocol communications: M1 (gateway modules), M2 (temperature sensors), and M3 (actuator nodes). They are responsible of data measurement and collection [32].

Besides, level 1 includes a real time controller of the energy capture at the solar collector, the thermal heat storage in the underground system, and the dynamic thermal barrier control.

The communications channels via Ethernet port connect user and data visualization nodes (mobile interface). A gateway CAN-bus port connects the perceptual and action nodes. Information transferring to databases, and shared memory update are also built-in functionalities at this low-level controller.

**Figure 11:**
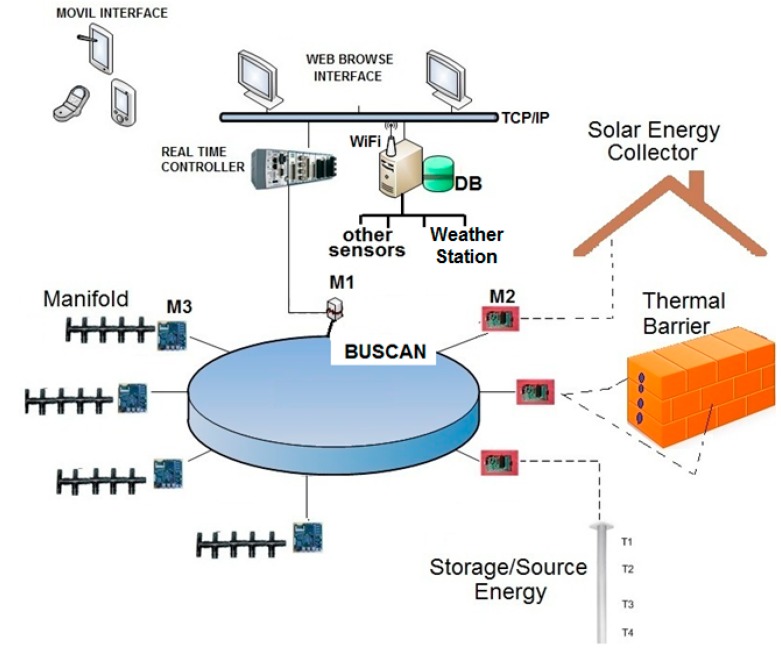
Level 1: Sensor and communication layer.

Figure 11 shows the main elements of this bottom level.

- M1: IoT gateway module.
- M2: IoT nodes dedicated to monitoring temperatures at different locations: indoor temperature (Ti), subsurface probes (Ts), walls (TW), and windows (Tw).
- M3: IoT actuator nodes, acting on the valves and monitoring the volumetric flow rate. They measure both fluid and roof's faces temperatures and provide web service functionalities through a TCP/IP stack and a light web server. The embedded web pages are stored in low resolution, to support commands and exchange information with end users.
- Real Time Controller (RTC): main control system with VxWorks operating system, and web server.
- Workstation and Data Base (DB): workstation is mainly used to exchange data and information with the Real Time Controller (RTC) but also to get data from other sensors. A DB is used to store data for further processing.
- Other sensors, mainly presence sensors.
- Weather station: weather variables such as solar irradiation and outdoor humidity.
- Mobile and Web Interface: the RTC and M3 IoT actuator nodes have a Web Server to show information by opening a web browser on mobile applications.

**Figure 12:**
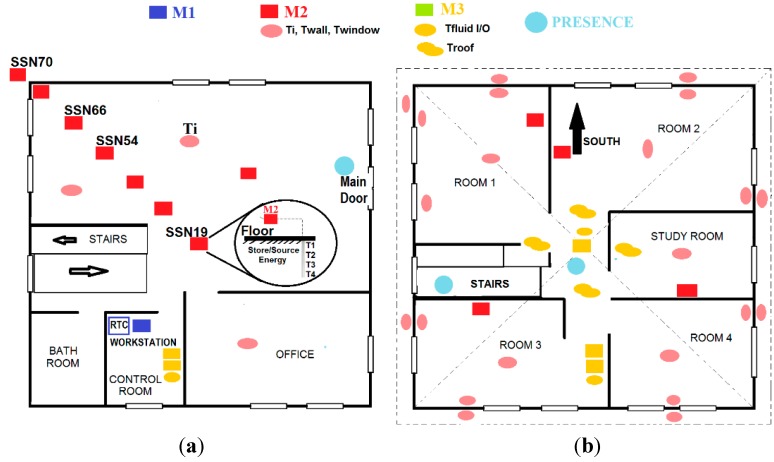
IoT nodes distribution in the nZEB. (**a**) Lower floor; (**b**) Upper floor.

The distribution of the IoT nodes and processors in both, the lower and the upper floor of the nZEB building is shown in Figure 12. In the lower floor, the RTC and the workstation are located in the lower floor (control room), with the gateway module, M1, that connects the CAN-BUS to the RTC.

**Figure 13:**
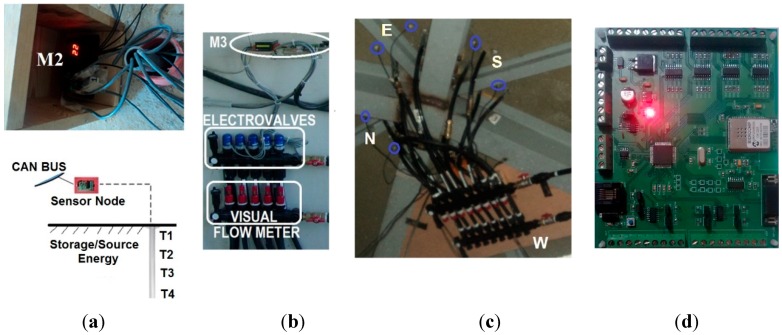
(**a**) M2 IoT node for underground temperature measurement; (**b**) M3 IoT node to control the opening/closing of the dynamic thermal barrier electro-valves; (**c**) Electro-valves at the ceiling; (**d**) M3 IoT sensor node.

On the lower floor, M2 type IoT nodes (red square) are located at different ground depths (T1, T2, T3, and T4) to measure underground temperature (Figure 12a). Another M2 sensor (red circle) measures indoor temperature (Ti). On the upper floor, the M2 nodes measure indoor, walls and window temperatures (Figure 12b). The M3 IoT nodes (yellow square) control the pumps (on/off) and the electro-valves (open/close) to let the fluid flow from the roof to both the ground heat exchanger and to the dynamic thermal barrier. The M3 node has built-in webserver capabilities, being an instance of a new generation sensor web enablement [33,34]. Presence sensors (blue circles) are located on both floors. Figure 13 shows some of the real sensors and actuators that have been installed in the nZEB building.

**Figure 14:**
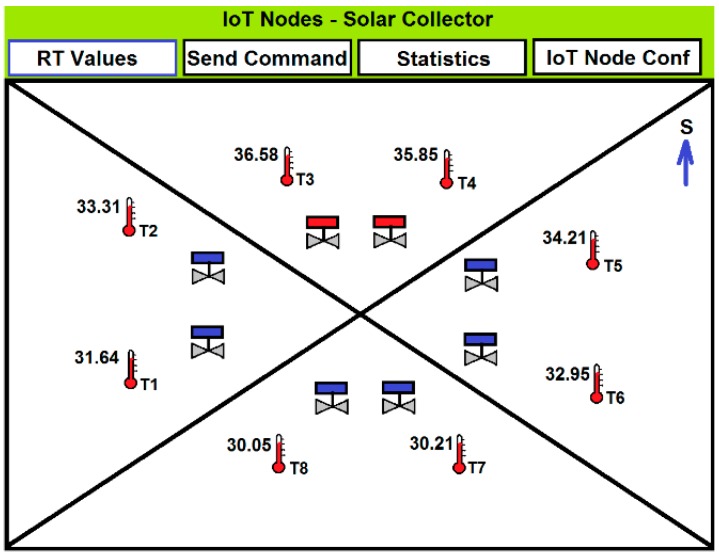
M3 type IoT node light web server screen.

A low resolution light webserver screen, that corresponds to a M3 node of the solar collector is presented in Figure 14, where real-time sensor values are shown. Besides, users have access to the last 48 hours statistical data, and to the IoT node configuration (ID, location, sampling rate). Moreover, users can send commands from this node, such as directing the thermal flow from the solar collector to the dynamic thermal barrier*.*

### 3.2. Level 2: User and Building Information Layer

At this level of the architecture, two main elements are considered: a fuzzy logic controller and virtual sensors. On the one hand, the thermal energy flow is qualitatively modelled by linguistic terms due to the imprecision inherent to the values of the variables involved in the system. Therefore, the control action that calculates the fluid flow to feed the dynamic thermal barrier is obtained by a fuzzy controller. Fuzzy logic exploits imprecision and uncertainty, and provides simple and low-computational cost solutions. On the other hand, virtual sensors have been designed and implemented to monitor the thermal barrier and to include context information and user preferences.

#### 3.2.1. The Fuzzy Controller

**Figure 15:**
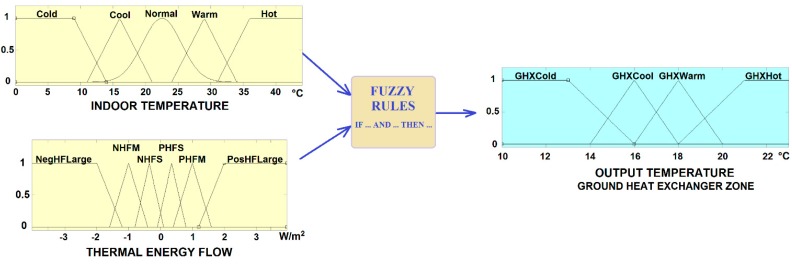
Fuzzy controller for the selection of the thermal energy source zone.

The fuzzy logic based controller has been designed to determine the thermal energy zone of the ground heat exchanger that has to be selected in order to reach the desired temperature at the thermal barrier (Figure 15).

The inputs of the fuzzy system are the indoor temperature, ranging from 0 °C to 44 °C, and the thermal energy flow ranging from −5 to 5 W/m^2^. Negative values mean that the direction of the flow may be in opposite direction (in cold days). Five linguistic terms are assigned to the indoor temperature: {Cold, Cool, Normal, Warm, Hot}, represented by Gaussian, triangular and trapezoidal membership functions. The other input, the thermal energy flow, has six fuzzy sets described by triangular and trapezoidal membership functions, labelled: {Negative Heat Flow Large (NegHFLarge), Negative Heat Flow Medium (NHFM), Negative Heat Flow Small (NHFS), Positive Heat Flow Small (PHFS), Positive Heat Flow Medium (PHFM), Positive Heat Flow Large (PosHFLarge)}.

The output variable is the desired output temperature at the thermal barrier, which is mapped to the corresponding ground heat exchanger zone (GHX) that has to send the heat flow. Five triangular and trapezoidal fuzzy sets: {GHXCold, GHXCool, GHXWarm, GHXHot} are distributed in the interval [10–22 °C].

The fuzzy knowledge base is presented in Table 3. Usually, the fluid flows through the walls in periods of less than 6 min. Input and output temperatures at the manifolds are checked to confirm that the thermal energy is dispatched.

**Table 3:** Fuzzy Knowledge Base.

Temperature/Thermal Flow	NegHF Large	NHFM	NHFS	PHFS	PHFM	PosHF Large
COLD	GHXHot	GHXWarm	GHXCool	GHXCold	GHXCool	GHXHot
COOL	GHXHot	GHXHot	GHXHot	GHXWarm	GHXWarm	GHXCool
NORMAL	GHXCool	GHXWarm	GHXHot	GHXHot	GHXHot	GHXWarm
WARM	GHXCool	GHXWarm	GHXHot	GHXHot	GHXHot	GHXHot
HOT	GHXCold	GHXCold	GHXCool	GHXWarm	GHXCool	GHXCold

#### 3.2.2. Thermal Barrier and Home and User Context Virtual Sensors

Virtual sensor integrate multiple and heterogeneous information, such as expert and historical knowledge, user profiles, and data from physical sensors and actuators. They can be defined as a tuple (Equation (12)):

(12) $$\text{VSi }=\left\{<i_{1},\;\ldots ,\;i_{N}>,\;<d_{1},\;\ldots ,\;d_{N}>,\;<O_{1},\;\ldots ,\;O_{j}>,\;<c_{1},\;\ldots ,c_{k}>\right\}$$

where *i_N_* denotes the set of inputs, *d_N_* the set of sensor raw data values, *O_j_* the set of outputs, and *c_k_* additional data about virtual sensor configuration. This virtual sensor structure allows to process raw data adding semantics information and also to build context information for later use.

In this work, two virtual sensors are proposed: Thermal Barrier and Home&User Context. The weather conditions (air temperature, solar radiation, wind, rain), building variables (indoor temperature and user presence), measured by physical sensors at level 1, and user information (profile/agenda) are the sources of the information of the virtual sensors of our smart building.

a) Thermal Barrier Virtual Sensor

The Thermal Barrier Virtual Sensor (TBVS) includes two elements: the fuzzy controller (Section 3.2.1) and building information (wall and indoor temperature of first and second floor, and thermal energy flow through the envelope). The fuzzy controller selects the ground heat exchanger zone, accordingly to the indoor temperature and the thermal energy flow on the exterior walls. These modules are part of the main real time application running at the central processor.

**Figure 16:**
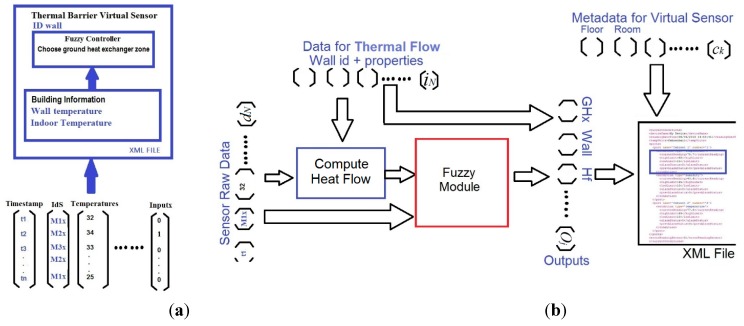
Thermal Barrier Virtual Sensor. (**a**) Inputs; (**b**) Components and data flow.

Besides, additional information such as wall properties or thermal fluid flow characteristics is structured as an XML file and introduced in the virtual sensor, so that the TBVS not only integrates raw data but also semantic information (Figure 16a). The data flow is shown in Figure 16b, where sensor raw data and wall properties are used to compute heat flow.

b) Home and User Context Virtual Sensor

**Figure 17:**
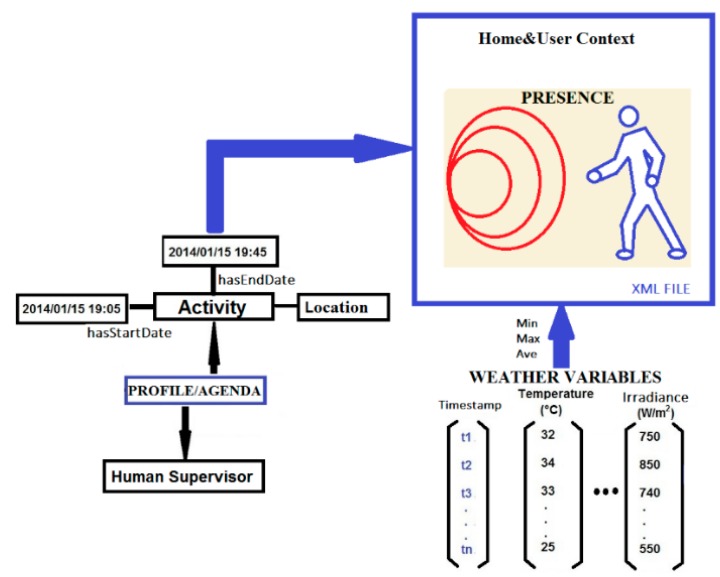
Home&User Context Virtual Sensor.

The Home&User Context Virtual Sensor (H&UC) processes information from three sources, Figure 17. The first one is the user’s profile and agenda, with information concerning location and beginning/end time of user tasks. This information allows planning ahead the control of the thermal flow. Second, the presence sensor provides information on the user location. In this case, the focus is on the second floor where two sensors are strategically located to act on the dynamic thermal barrier. Third, weather conditions such as solar irradiation or outdoor air temperature.

### 3.3. Level 3: Decision Layer

The top layer of the three-level proposed architecture uses a parser to extract information from the virtual sensors and to make a decision on the activation of the dynamic thermal barrier. In this case, a decision tree with a few rules is applied to determine whether the thermal barrier must be activated and the zone of the heat exchanger to send the thermal flow.

**Figure 18:**
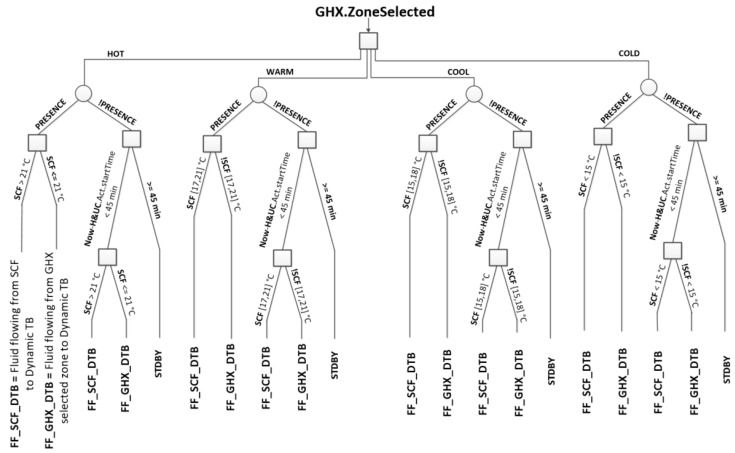
The decision tree designed to activate the dynamic thermal barrier.

The decision tree (Figure 18) root node has the output of the fuzzy controller as the root node, *i.e.*, the ground heat exchanger zone selected. Let’s explain the left brunch of the decision tree. The node at the second level of the tree is the ground heat exchanger zone, in this case GHX.ZoneSelected = “HOT”. If the presence sensor is activated and the solar collector temperature (SCF) is greater than 21 °C then the conclusion is “FF_SCF_DTB”, meaning that the fluid must flow through the PP pipes from the solar collector to the dynamic thermal barrier. Otherwise, if SCF < 21 °C, the outcome is “FF_GHX_DTB”, which means that the fluid flows through the PP tubes from the ground heat exchanger zone selected (HOT) to the dynamic thermal barrier.

Whenever any presence sensor is activated, the temperature at each roof face (north, south, east and west) of the solar collector is checked to determine whether or not it can supply the thermal energy demanded.

If the presence sensor is not activated then the “home and user context” virtual sensor is switch on and checks if any activity programmed by the user starts in less than 45 min (Now–H&UC.Act.startTime). If there is any, and SCF is greater than 21 °C, the thermal barrier will be activated (“FF_SCF_DTB”) using the solar collector to send the thermal fluid to the dynamic envelope. Otherwise, “FF_GHX_DTB” is activated to send the fluid flow from the ground heat exchanger zone selected. Otherwise the dynamic thermal barrier is not activated.

Remark that the solar collector temperatures are sampled each 15 min. Usually in summer and during the daylight time, the south collector thermal energy flow is exclusively led to the hot zone of the ground heat exchanger.

## 4. Experimental Results and Discussion

Real measurements have been obtained from the experiments carried out in the nZEB building located at Arganda del Rey, Madrid (Spain) shown in Figure 1. The tests performed on the thermal components of the building: low enthalpy geothermal system, solar energy collector, ground heat (thermal) exchanger, and dynamic thermal barrier, have proved the efficiency of the proposed perception and control architecture.

**Figure 19:**
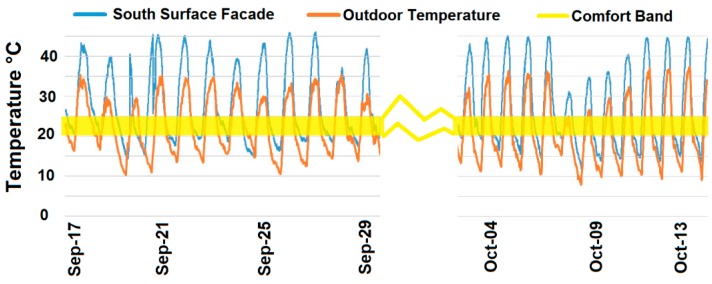
Temperatures in the inner South surface of the roof, mid-September to mid-October.

The roof solar collector shows a high absorptivity and, consequently, the water-glycol solution in the PP tubes reaches average values close to 65 °C during summer daylight, and around 25 °C in winter sunny days. Temperatures measured at its inner South surface are represented in Figure 19. These values were collected during the autumn, where average outdoor temperature is medium. Yellow line in Figure 19 corresponds to the indoor building thermal comfort band.

**Figure 20:**
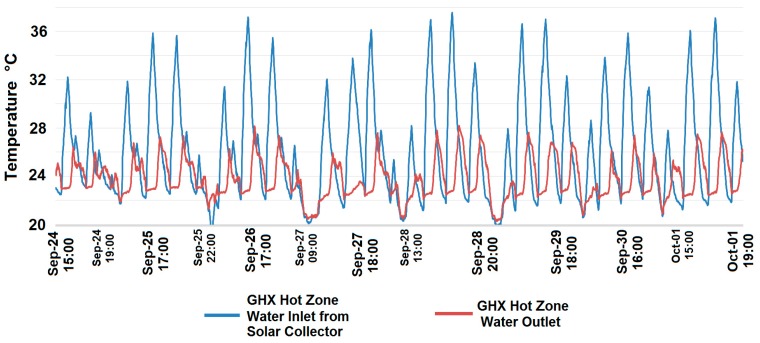
Temperature at the water inlet and outlet sensors of the ground heat exchanger.

Temperatures given by the water inlet and outlet sensors of the ground heat exchanger are presented in Figure 20. They show that at the end of September the temperature reaches values above 35 °C and, as a result, there were two possibilities of transferring the thermal energy according to the decision tree: to the dynamic thermal barrier for direct use, or as it happened in this case, to one of the ground heat exchanger zones where it was stored for later use.

**Figure 21:**
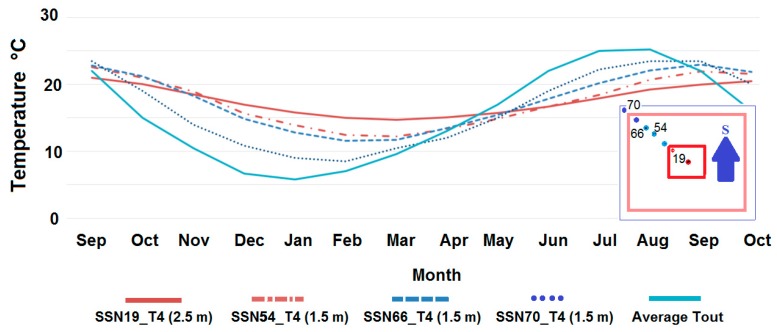
Underground soil temperature along a year.

Ground heat exchanger temperatures for the hot zone (SSN19_T4), cool zone (SSN54_T4 and SSN66_T4), and cold zone (SSN70_T4), are represented in Figure 21. The square box at the right-bottom of that figure shows the underground sensor locations. The graphic shows the attenuation of the influence of external conditions on the ground temperature due to its own thermal inertia. The least influenced zones are those closer to the centre of the building. Therefore, the zone most affected by the weather conditions is the cold one, and the least affected the hot zone.

The temperature variations at the different zones of the ground reveal the soil thermal inertia. This property can be used to store energy in the soil, reducing the thermal losses in the envelope and, therefore, decreasing the energy demand (heating/cooling). At the same time, this fact highlights the possibility of controlling the underground temperature by zones, independently of the external conditions. This process is always limited by the capability for capturing the thermal energy (heating/cooling).

**Figure 22:**
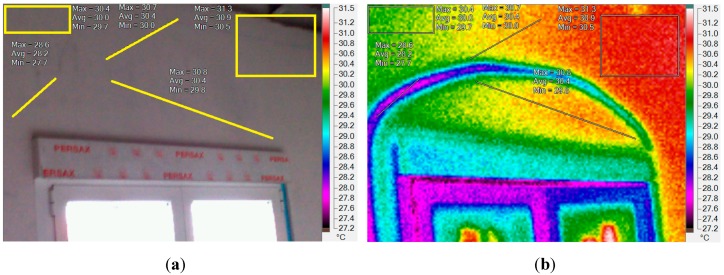
(**a**) Visual image of a wall and window in grey scale; (**b**) Thermal image with the fluid flowing through the wall barrier PP tubes, in false colour.

A thermographic analysis has been also carried out in the South indoor face of the building (Figure 1b,c) where the thermal barrier has been implemented. The visual image in grey-scale and a false-colour thermal image of the South indoor face and window, when the dynamic thermal barrier is activated, are displayed in Figure 22a,b, respectively. They have been taken in August at 10 am, and the fluid was flowing from the roof solar collector for about 4 min, being the inlet temperature 27.5 °C. This temperature allows the fluid to flow to the hot zone where the thermal energy (heat) is stored. Before the fluid starts flowing, the temperature in the wall was 30.5 °C.

At the left side of the figures, the values of the temperatures are: Max = 28.6 °C, Avg = 28.2 °C, and Min = 27.7 °C. These values are lower than the ones measured far from the pipe. That behaviour shows how the refrigeration effect spreads according to the distance to the tubes. The thermal image (Figure 22b) shows how the dynamic thermal barrier smoothers the inner surface temperature, and therefore, reduces the energy demand.

Consequently, it is possible to transfer the thermal energy flow from the roof solar collector either to the dynamic barrier of the walls, to change the temperature gradient between outdoor and indoor, or to the underground storage zones of the low geothermal enthalpy system.

## 5. Conclusions

Three thermal systems have been designed, modelled and implemented in a near Zero-Energy Building prototype. The main goal of this infrastructure is to meet the energy demand of the occupants while making a better use of the thermal energy available.

A solar collector has been implemented in the roof to capture energy. This thermal system is connected to a low geothermal enthalpy system, designed to store the solar thermal energy, and to a dynamic envelope. This novel proposal of thermal barrier is composed of a set of pipes embedded in concrete layer of the walls, between two PP isolation layers. This element has been proved to be efficient reducing the thermal energy losses and helping to stabilize the inner building temperature within the comfort band.

Besides, a perception and control architecture is proposed in order to control the fluid flow between the thermal systems. This three layers configuration includes a sensor and communication network, the definition of virtual sensors that include semantic information of the user behaviour and of the building characteristics, and a decision-making system based on a decision tree.

Simulation and real experiments on the building shows the benefits of controlling the flow between those thermal systems. Indeed, the dynamic thermal barrier allows to modify the thermal energy transfer flow (heat or cold) and, therefore, to change the wall inner face temperature at an affordable cost, resulting in a decrease in the thermal energy consumption.

Future work will include the relative humidity in the comfort zone definition because as it plays a significant role in humid regions. In this paper, only indoor dry bulb temperature has been considered. Other future development would be the integration of adaptive capabilities to the fuzzy controller to face the changes in the soil temperature over a year and to apply predictive control.
